# Systematic review and meta-analysis of lung cancer brain metastasis and primary tumor receptor expression discordance

**DOI:** 10.1007/s12672-021-00445-2

**Published:** 2021-11-08

**Authors:** Raees Tonse, Muni Rubens, Haley Appel, Martin C. Tom, Matthew D. Hall, Yazmin Odia, Michael W. McDermott, Manmeet S. Ahluwalia, Minesh P. Mehta, Rupesh Kotecha

**Affiliations:** 1 Department of Radiation Oncology, Miami Cancer Institute, Baptist Health South Florida, Office 1R203, Miami, FL 33176 USA; 2 Office of Clinical Research, Miami Cancer Institute, Baptist Health South Florida, Miami, FL USA; 3 Herbert Wertheim College of Medicine, Florida International University, Miami, FL USA; 4 Division of Neuro-Oncology, Miami Cancer Institute, Baptist Health South Florida, Miami, FL USA; 5 Department of Neurosurgery, Miami Neuroscience Institute, Baptist Health South Florida, Miami, FL USA; 6 Department of Medical Oncology, Miami Cancer Institute, Baptist Health South Florida, Miami, FL USA

**Keywords:** EGFR, KRAS, Receptor, Discordance, Metastasis, Brain

## Abstract

**Background:**

Treatment paradigms for metastatic non-small cell lung cancer are increasingly based on biomarker-driven therapies, with the most common alteration being mutation in the epidermal growth factor receptor (EGFR). Change in expression of such biomarkers could have a profound impact on the choice and efficacy of a selected targeted therapeutic, and hence the objective of this study was to analyze discordance in EGFR status in patients with lung cancer brain metastasis (LCBM).

**Methods:**

Using PRISMA guidelines, a systematic review was performed of series in the Medline database of biopsied or resected LCBM published before May, 2020. Key words included “lung cancer” and “brain metastasis” combined with “epidermal growth factor receptor/EGFR,” and “receptor conversion/discordance or concordance.” Weighted random effects models were used to calculate pooled estimates.

**Results:**

We identified 501 patients from 19 full-text articles for inclusion in this study. All patients underwent biopsy or resection of at least one intracranial lesion to compare to the primary tumor. On primary/LCBM comparison, the weighted pooled estimate for overall EGFR receptor discordance was 10% (95% CI 5–17%). The weighted effects model estimated a gain of an EGFR mutation in a brain metastases in patients with negative primary tumors was 7% (95% CI 4–12%). Alternatively, the weighted effects model estimate of loss of an EGFR mutation in patients with detected mutations in the primary tumor was also 7% (95% CI 4–10%). KRAS testing was also performed on both primary tumors and LCBM in a subset of 148 patients. The weighted effects estimate of KRAS-mutation discordance among LCBM compared to primary tumors was 13% (95% CI 5–27%). The weighted effects estimated of KRAS gain and loss in LCBM was 10% (95% CI 6–18%) and 8% (95% CI 4–15%), respectively. Meta-regression analysis did not find any association with any factors that could be associated with discordances.

**Conclusions:**

EGFR and KRAS mutation status discordance between primary tumor and LCBM occurs in approximately 10% and 13% of patients, respectively. Evaluation of LCBM receptor status is key to biomarker-driven targeted therapy for intracranial disease and awareness of subtype switching is critical for those patients treated with systemic therapy alone for intracranial disease.

**Supplementary Information:**

The online version contains supplementary material available at 10.1007/s12672-021-00445-2.

## Introduction

Lung cancer remains a leading cause of cancer death worldwide with more than 50% of patients diagnosed with advanced stage disease at initial diagnosis [1]. An increased understanding of molecular pathology over the past decades has advocated personalized treatment approaches. Molecular diagnostic testing is now recommended in clinical guidelines for all lung cancer patients to determine the eligibility for targeted therapies [2]. Epidermal growth factor receptor (EGFR) mutation is one of the most common actionable mutations and predictive of treatment response to tyrosine kinase inhibitors (TKIs) [3, 4]. Patients with EGFR mutations in exon 19 and 21 have longer median progression-free survival (PFS) than those with wild-type EGFR disease (15.2 months versus 4.4 months) [5].

With the increasing availability of TKIs with intracranial penetration, the prognosis of brain metastasis (BM) lung cancer patients harboring EGFR mutations has improved. Decisions regarding the use of EGFR-directed therapies are typically made based on molecular testing of the initial tumor pathology or via liquid (blood) biopsy. Given the recent understanding of the potential discordance between primary tumors and brain metastasis [6, 7], the objective of this study was to analyze discordance in EGFR status in patients with lung cancer brain metastasis (LCBM). A well-conducted systematic review and meta-analysis focusing on the discordance rate of EGFR mutation status between primary tumor and brain metastases has not been published and would be timely.

## Methods

### Selection of articles

The Preferred Reporting Items for Systematic Reviews and Meta-Analyses (PRISMA) criteria were used to conduct this systematic review of the literature [8]. This review has been registered on PROSPERO (ID: CRD42021272056).

MEDLINE (PubMed) (https://pubmed.ncbi.nlm.nih.gov/) and the CENTRAL (Cochrane Central Register of Controlled Trials) (https://www.cochrane.org/) electronic bibliographic databases were used to screen for the initial articles. Additional research studies were included based on an assessment of the selected article bibliographies and other literature reviews. Key words used during the initial search strategy included “lung cancer” and “brain metastasis” combined with “epidermal growth factor receptor/EGFR,” and “receptor conversion/discordance or concordance”. The search strategy used for both the database is listed in supplemental Table 1. Full text publication in the English language published up through April 2021 were evaluated. The screening of articles was done manually.

During the initial search, the PICOS (Population, Intervention, Control, Outcomes, Study Design) methodology (supplemental Table 2) was used to determine the inclusion criteria. The initial search yielded 992 publications which were then screened by careful review of the article titles, abstracts and manuscripts. Original full-text research publications, retrospective or prospective case series of > 10 adult patients documenting EGFR and KRAS status in primary lung cancers compared to LCBM, and receptor conversion or discordance were all considered as inclusion criteria. Non-clinical studies, expert opinion, commentary, research with data on fewer than ten patients, and studies on patients with lung cancer that only compared receptors to extracranial metastases were excluded. Publications in other languages besides English and those available only in abstract form were excluded. A manual review of the references of retrieved articles was performed to locate additional relevant publications. Duplicate studies were checked for any new in-formation, and the most recent report with the greatest number of patients was included in the final analysis. The search strategy used for this report and the methodology for study inclusion is illustrated in supplemental Fig. 1.

The study details abstracted for this analysis included year of publication, single center or multi-institutional study, the duration of the study period, the number of patients included, median age, sex (male/female), smoking status (smoker/never smoker), and histology (NSCLC/SCLC). The number of LCBM was evaluated in each study and divided in three categories: 1, 2–5, and > 5. Median brain metastasis-free interval was also documented. Diagnostic and therapeutic interventions for the brain metastasis, including biopsy or resection, stereotactic radiosurgery (SRS), whole brain radiotherapy (WBRT), targeted therapy (i.e., geftinib, erlotinib, etc.), and immunotherapy use was also noted.

The techniques for determining EGFR and KRAS status were included. The EGFR mutation status included hotspot regions in exons 18, 19, 20 and 21 at initial diagnosis of the primary tumor and of the LCBM. The KRAS mutation status of primary tumor and the brain metastases was also documented. Data on receptor discordance included LCBM to primary tumor discordance based on EGFR and KRAS mutation status. A change in mutation status from mutant to wild-type or vice versa was defined as discordance. For this analysis, a change in one EGFR mutation to a different EGFR mutation was not considered discordant. Gain or loss of EGFR and KRAS status were also recorded. Grading of Recommendations, Assessment, Development and Evaluation (GRADE) approach was used to assess quality of the body of evidence (supplemental Table 3) [9].

### Outcome measures and statistical analysis

The individual receptor status of the primary tumor and LCBM was documented. The receptor discordance data included the LCBM to primary tumor discordance based on individual receptor expression (gain or loss of each individual receptor). For the meta-analyses, R (version 1.1.423, Boston, Massachusetts) was used with R package “metafor” (version 2.0–0) [10]. DerSimonian-Laird method was used for calculating study variances for overall estimates [11]. For each of outcome variable, weighted random effects models were used to calculate pooled estimates. The random effects models were used for calculating pooled estimates because of the heterogeneity of studies included in the analyses [12]. The I^2^ statistic was used to determine heterogeneity with 0%, 25%, 50%, and 75% interpreted as absent, low, moderate, and high heterogeneity, respectively. For detecting publication bias, funnel plots and the Egger test (P value < 0.05 indicating presence of bias) were utilized. Finally, meta-regression analysis was performed to determine if factors like age, sex, smoking status, and histology were associated with receptor expression discordance.

## Results

We identified 19 full-text articles on 501 patients that contained EGFR expression analyses and met the inclusion criteria for this study. All patients had at least one intracranial lesion biopsy or excision. No publication bias (p > 0.05) was detected across the included reports regarding the primary outcomes evaluated in this study (see supplemental Figs. 2 and 3). All included studies were retrospective in nature and considered low-quality evidence. A majority of studies (n = 16, 84%) represented single-institution reports, and three (16%) were multi-institutional collaborations. Each study had a median of 15 patients (range: 3–143 people) (see Table 1). The literature did not report key patient features, demographics, or therapeutic information in a uniform or consistent manner. Across all studies, 72% were male, and 67% patients reported positive smoking history. The median age was 57 years (range: 52–66 years) and the patients diagnosed with histology NSCLC and SCLC were 87% and 13% respectively. The time interval between primary tumor and development of LCBM was 16 months (range 3–30 months). The number of lesions at brain metastasis diagnosis was not reported in most studies.

**Table 1:** Lung cancer brain metastases study details and patient characteristics

Author	Year	Institution	Years	Evidence quality	N	Median Age	Sex		Smoking status		Histology		# Brain metastasis at BM Dx			Median BM free interval (months)	Resection	SRS	WBRT	Targeted therapy		Immunotherapy
							Male	Female	Smoker	Never smoker	NSCLC	SCLC	1	2 to 5	> 5					Geftinib	Erlotinib	
Matsumoto et al. [37]	2006	Single-centre	1986–2001	Low	19	52	13	6	13	6	19	0	NA	NA	NA	NA	19	NA	NA	0	NA	NA
Italiano et al. [38]	2006	Single-centre	1990–2003	Low	20	57	16	4	15	5	16	4	NA	NA	NA	9	20	NA	NA	NA	NA	NA
Takahashi et al. [39]	2007	Single-centre	NA	Low	7	55	4	3	NA	NA	4	3	NA	NA	NA	NA	7	NA	NA	NA	NA	NA
Kalikaki et al. [40]	2008	Single-centre	NA	Low	3	55	2	1	3	0	3	0	NA	NA	NA	30	3	NA	NA	1	NA	NA
Gow et al. [41]	2009	Single-centre	1996–2004	Low	25	61	NA	NA	NA	NA	23	2	NA	NA	NA	9.3	25	3	1	0	NA	NA
Daniele et al. [42]	2009	Multi-centre	2004–2006	Low	28	66	23	5	NA	NA	21	7	NA	NA	NA	NA	28	NA	NA	0	0	NA
Cortot et al. [43]	2010	Single-centre	1990–2003	Low	13	60	15	6	NA	NA	16	3	NA	NA	NA	NA	13	NA	NA	0	0	NA
Han et al. [44]	2011	Multi-centre	1997–2010	Low	5	NA	4	1	3	2	NA	NA	NA	NA	NA	NA	5	NA	NA	2	0	NA
Fang et al. [45]	2011	Single-centre	NA	Low	4	NA	NA	NA	NA	NA	4	0	NA	NA	NA	NA	4	NA	NA	NA	NA	NA
Munfus-McCray et al. [46]	2011	Single-centre	2007–2010	Low	10	60	NA	NA	NA	NA	NA	NA	NA	NA	NA	18.3	9	3	2	2	5	NA
Grommes et al. [47]	2011	Single-centre	NA	Low	9	57	2	7	NA	NA	NA	NA	NA	NA	NA	3.3	1	1	2	0	4	0
Kamila et al. [48]	2013	Single-centre	2003–2010	Low	143	59	99	44	111	32	120	23	NA	NA	NA	NA	143	NA	NA	NA	NA	NA
Luo et al. [49]	2014	Single-centre	2007–2012	Low	15	55	NA	NA	NA	NA	NA	NA	NA	NA	NA	13.7	136	13	42	7	NA	NA
Quere et al. [50]	2016	Single-centre	2005–2012	Low	7	61	NA	NA	NA	NA	NA	NA	NA	NA	NA	NA	44	NA	NA	NA	NA	NA
Rau et al. [51]	2016	Single-centre	1991–2010	Low	49	63	27	22	26	18	NA	NA	NA	NA	NA	NA	44	NA	NA	14	NA	NA
Liao et al. [30]	2018	Single-centre	NA	Low	6	53.5	5	1	0	6	6	0	NA	NA	NA	16	6	NA	NA	NA	NA	NA
Kobayashi et al. [31]	2018	Single-centre	1985–2014	Low	59	61	45	14	40	19	NA	NA	31	28		19.2	59	14	24	2	0	0
Kim et al. [32]	2019	Single-centre	2011–2016	Low	18	63	11	7	7	11	NA	NA	NA	NA	NA	NA	18	NA	NA	5	3	NA
Wang et al. [16]	2019	Multi-centre	2000–2016	Low	61	57	43	18	30	31	61	0	NA	NA	NA	22.6	60	NA	NA	5		0

N: Number; NSCLC: Non-small cell lung cancer; SCLC: Small cell lung cancer; BM: Brain metastasis, SRS: Stereotactic radiosurgery; WBRT: Whole brain radiotherapy; NA: Not available

Details regarding EGFR and KRAS mutation status of primary tumor and at the time of brain metastasis are presented in Table 2. The mutation assessment technique varied across the studies, some included direct sequencing, IHC > 10% and FISH, high-resolution SNP array, RT-PCR analysis, ARMS method, whole exome sequencing and targeted panel sequencing, and next-generation sequencing. The EGFR mutation status at initial diagnosis of the primary tumor showed EGFR mutant in 149 patients and EGFR wild type in 347 patients and LCBM showed EGFR mutant 134 patients and EGFR wild type in 311 patients. Hotspot regions in exon 18, exon 19, exon 20, and exon 21 were found to be 5 patients, 60 patients, 1 patient, and 54 patients for primary tumor and 3 patients, 65 patients, 2 patients, and 40 patients for LCBM, respectively. EGFR mutation status were most commonly seen in exons 19 and 21 for both primary tumour and LCBM. The KRAS mutation status at initial diagnosis of the primary tumor showed a median number of KRAS mutations in 42 patients and KRAS wild type in 83 patients and LCBM with KRAS mutant in 42 patients and KRAS wild type in 26 patients.

**Table 2:** Lung cancer brain metastases EGFR and KRAS mutation status

Author	Year	N	Molecular marker assessment technique	EGFR mutation status of primary tumor						EGFR mutation status of brain metastases						KRAS status of primary tumor		KRAS status of brain metastases	
				EGFR mutant					EGFR wild type	EGFR mutant					EGFR wild type	KRAS mutant	KRAS wild type	KRAS mutant	KRAS wild type
				Exon 18	Exon 19	Exon 20	Exon 21	Total		Exon 18	Exon 19	Exon 20	Exon 21	Total					
Matsumoto et al. [37]	2006	19	Direct sequencing and Genomic PCR amplification	0	10	0	2	12	7	0	10	0	2	12	7	2	NA	2	NA
Italiano et al. [38]	2006	20	IHC > 10% and FISH	NA	NA	NA	NA	14	6	NA	NA	NA	NA	12	8	NA	NA	NA	NA
Takahashi et al. [39]	2007	7	High-resolution SNP array	NA	NA	NA	NA	0	7	NA	NA	NA	NA	0	7	NA	NA	NA	NA
Kalikaki et al. [40]	2008	3	Direct sequencing	NA	NA	NA	NA	1	2	NA	NA	NA	NA	1	2	2	1	3	0
Gow et al. [41]	2009	25	Direct sequencing and ARMS method	NA	NA	NA	NA	4	21	1	7	0	4	11	14	NA	NA	NA	NA
Daniele et al. [42]	2009	28	Direct sequencing	NA	NA	NA	NA	0	28	NA	NA	NA	NA	0	28	NA	NA	NA	NA
Cortot et al. [43]	2010	13	Direct sequencing and mutant-enriched PCR	NA	NA	NA	NA	0	13	NA	NA	NA	NA	0	13	1	1	1	1
Han et al. [44]	2011	5	Direct sequencing and Genomic PCR amplification	0	1	0	3	4	1	0	1	0	2	3	2	1	4	1	4
Fang et al. [45]	2011	4	RT-PCR analysis	0	0	0	1	1	3	NA	NA	NA	NA	1	3	NA	NA	NA	NA
Munfus-McCray et al. [46]	2011	10	RT-PCR analysis	NA	NA	NA	NA	4	2	NA	NA	NA	NA	4	2	4	0	4	0
Grommes et al. [47]	2011	9	RT-PCR analysis	1	4	0	4	9	0	0	1	0	3	4	0	NA	NA	NA	NA
Kamila et al. [48]	2013	143	DNA–FLA, ASP–PCR and PNA–LNA PCR clamp methods	0	3	0	6	9	134	0	1	0	1	2	134	NA	NA	NA	NA
Luo et al. [49]	2014	15	ARMS method	0	4	0	3	7	8	0	5	0	3	8	7	NA	NA	NA	NA
Quere et al. [50]	2016	7	RT-PCR analysis	0	0	0	1	1	5	0	0	0	1	1	5	2	4	2	4
Rau et al. [51]	2016	49	RT-PCR analysis	3	10	0	17	30	19	2	15	0	13	30	19	14	19	16	17
Liao et al. [30]	2018	6	Whole exome sequencing and targeted panel sequencing	0	3	0	1	4	2	0	3	0	1	4	2	NA	NA	NA	NA
Kobayashi et al. [31]	2018	59	RT-PCR analysis	0	8	0	6	14	45	0	3	0	2	5	15	5	54	NA	NA
Kim et al. [32]	2019	18	Real-time PCR clamping method	1	3	1	3	10	8	0	5	0	3	10	8	NA	NA	NA	NA
Wang et al. [16]	2019	61	Next-generation sequencing	0	14	0	7	25	36	0	14	2	5	26	35	11	NA	13	NA

N: Number; NA: Not available; IHC: Immunohistochemistry; FISH: Fluorescence in situ hybridization; RT-PCR: Reverse transcription polymerase chain reaction; EGFR: Epidermal growth factor receptor; KRAS: Kirsten rat sarcoma viral oncogene homolog

**Fig. 1:**
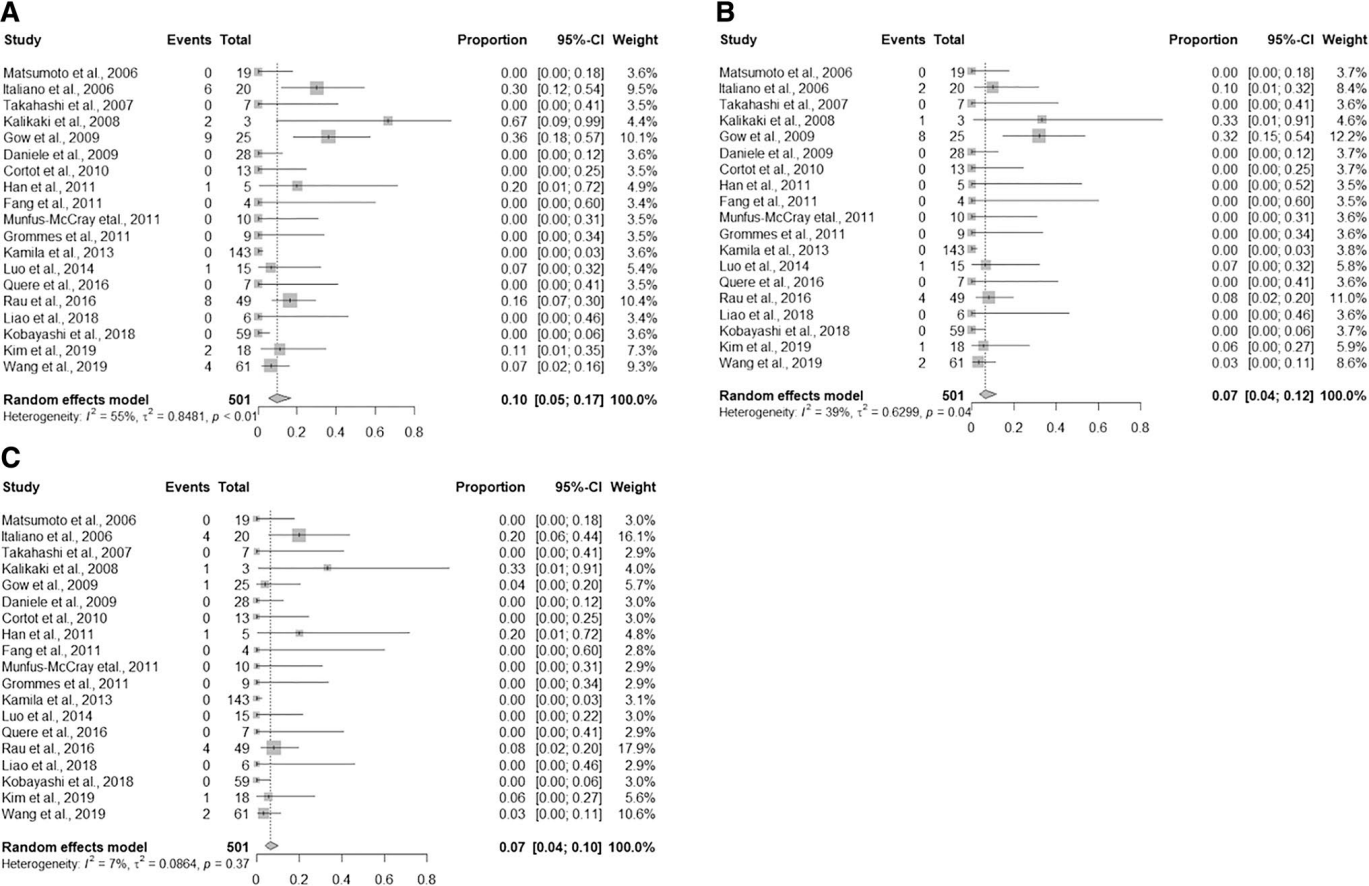
Forest plots of primary lung tumor and brain metastasis EGFR status. **A** Lung cancer/brain metastasis EGFR discordance, **B** BM EGFR gain, and **C** BM EGFR loss. In the forest plot, square box corresponds to proportions of individual study and horizontal line 95% confidence interval. Dimension of each box represent the weight of each study. The diamond represents pooled estimate with 95% confidence interval

**Fig. 2:**
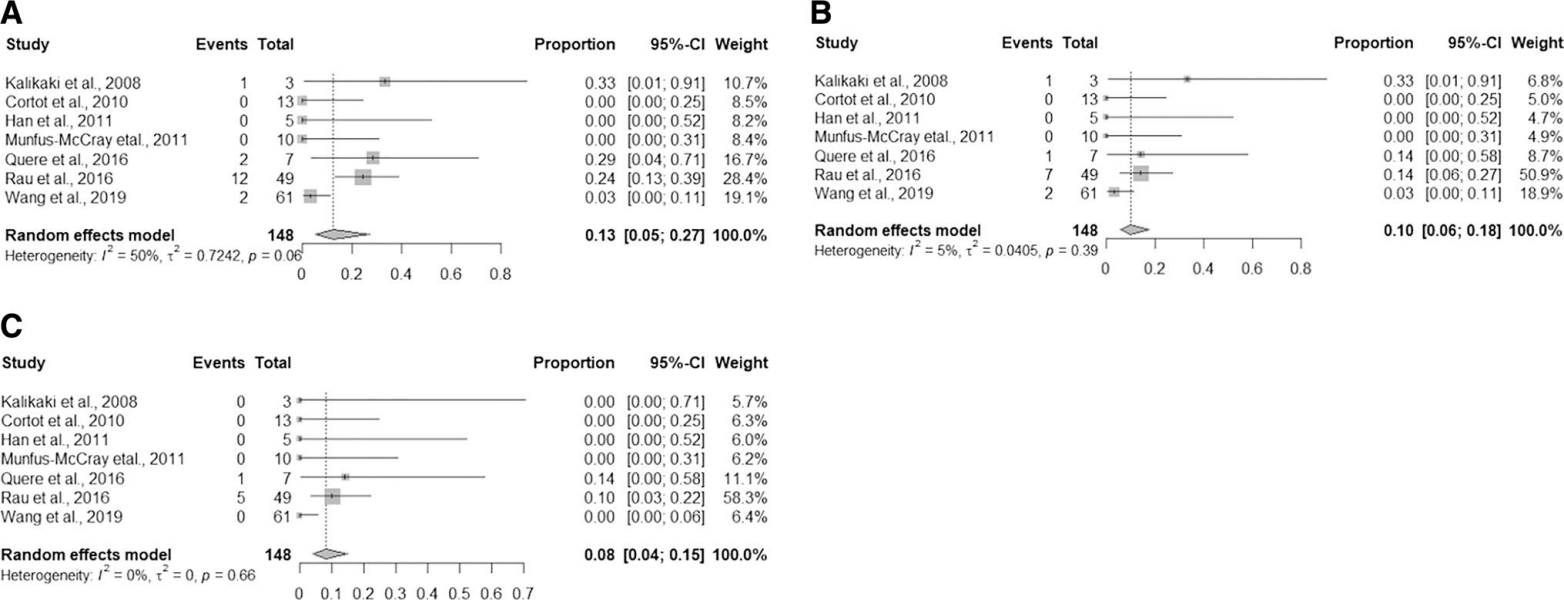
Forest plots of primary lung tumor and brain metastasis KRAS status. **A** Lung cancer/brain metastasis KRAS discordance, **B** BM KRAS gain, and **C** BM KRAS loss. In the forest plot, square box corresponds to proportions of individual study and horizontal line 95% confidence interval. Dimension of each box represent the weight of each study. The diamond represents pooled estimate with 95% confidence interval

**Fig. 3:**
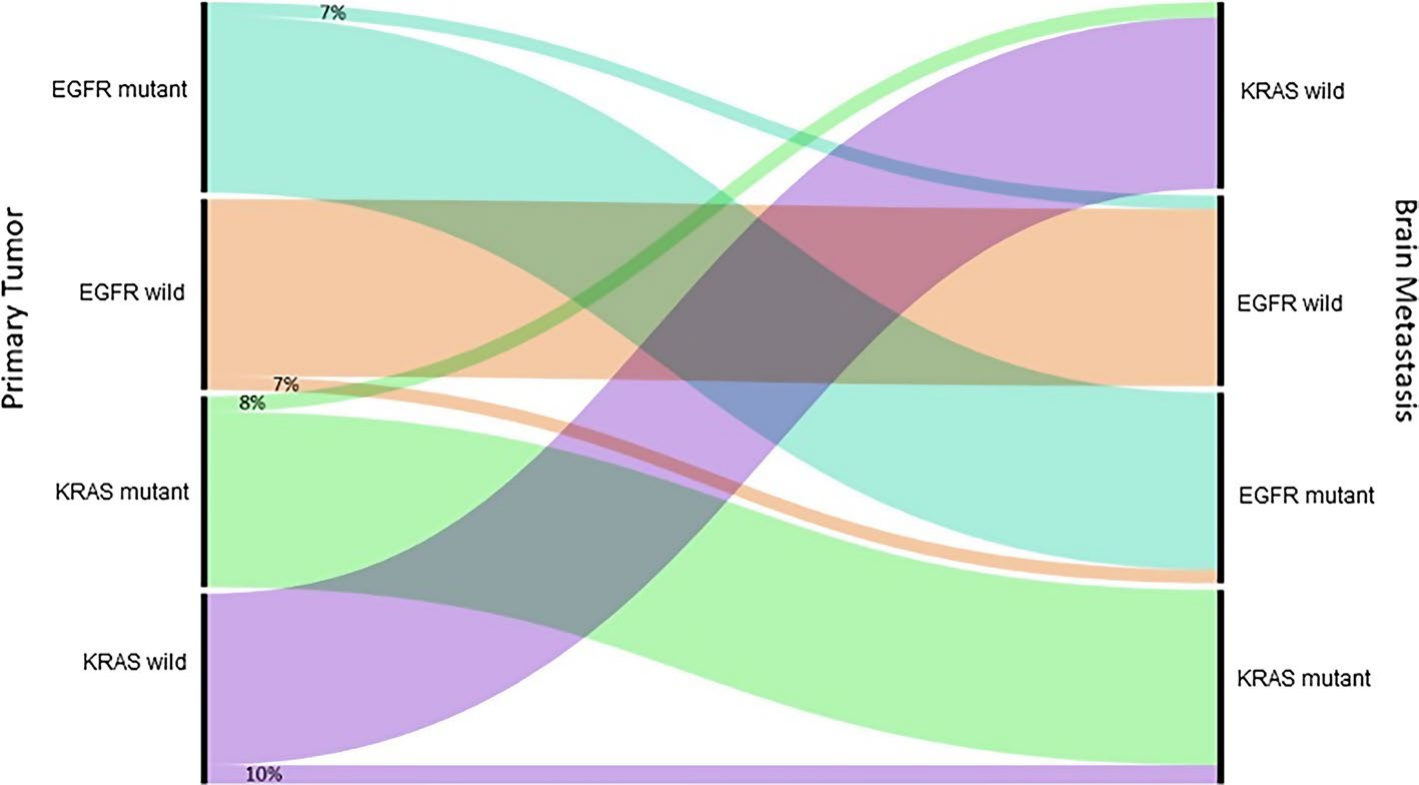
Alluvial diagram representing the receptor switch in EGFR and KRAS mutation between primary lung tumor and brain metastasis

On primary/LCBM comparison (see Table 3), the weighted pooled estimate for overall EGFR receptor discordance was 10% (95% CI 5–17%). The weighted effects model estimate of gain of an EGFR mutation in patients with negative primary tumors was 7% (95% CI 4–12%). Alternatively, the weighted pooled estimate of loss of an EGFR mutation in patients with detected mutations in the primary tumor was 7% (95% CI 4–10%) (see Fig. 1). KRAS testing was also performed on both primary tumors and LCBM in a subset of 148 patients. The weighted effects estimate of KRAS-mutation discordance among LCBM compared to primary tumors was 13% (95% CI 5–27%). The weighted effects estimated of KRAS gain in LCBM was 10% (95% CI 6–18%) and 8% (95% CI 4–15%) for KRAS loss (see Figs. 2 and 3). All mutation conversions were considered statistically significant (*p* < 0.05).

**Table 3:** Lung cancer with brain metastases mutation discordances

Author	Year	N	Lung/BM EGFR discordance	BM EGFR gain	BM EGFR loss	Lung/BM KRAS discordance	BM KRAS gain	BM KRAS loss
Matsumoto et al. [37]	2006	19	0	0	0	NA	NA	NA
Italiano et al. [38]	2006	20	6	2	4	NA	NA	NA
Takahashi et al. [39]	2007	7	0	0	0	NA	NA	NA
Kalikaki et al. [40]	2008	3	2	1	1	1	1	0
Gow et al. [41]	2009	25	9	8	1	NA	NA	NA
Daniele et al. [42]	2009	28	0	0	0	NA	NA	NA
Cortot et al. [43]	2010	13	0	0	0	0	0	0
Han et al. [44]	2011	5	1	0	1	0	0	0
Fang et al. [45]	2011	4	0	0	0	NA	NA	NA
Munfus-McCray et al. [46]	2011	10	0	0	0	0	0	0
Grommes et al. [47]	2011	9	0	0	0	NA	NA	NA
Kamila et al. [48]	2013	143	0	0	0	NA	NA	NA
Luo et al. [49]	2014	15	1	1	0	NA	NA	NA
Quere et al. [50]	2016	7	0	0	0	2	1	1
Rau et al. [51]	2016	49	8	4	4	12	7	5
Liao et al. [30]	2018	6	0	0	0	NA	NA	NA
Kobayashi et al. [31]	2018	59	0	0	0	NA	NA	NA
Kim et al. [32]	2019	18	2	1	1	NA	NA	NA
Wang et al. [16]	2019	61	4	2	2	2	2	0

N: Number; NA: Not available; BM: Brain metastasis; EGFR: Epidermal growth factor receptor; KRAS: Kirsten rat sarcoma viral oncogene homolog

We found no correlation between discordance and any factors in the meta-regression analysis.

## Discussion

Over the last few decades, advances in molecular biology in advanced lung cancer have allowed for more personalized treatment options. Clinical guidelines now suggest molecular diagnostic testing for patients with advanced NSCLC to establish a patient’s eligibility for targeted therapies [13]. In advanced NSCLC, one of the most prevalent actionable mutations is in the EGFR receptor, which predicts treatment response to TKIs, specifically with mutations in exons 19 and 21. Similarly, KRAS mutations are found in approximately 30% of NSCLC BM, and based on a recently completed phase I trial, the KRAS-targeted drug Sotorasib was recently approved [14]. As a result, this meta-analysis focusing on the rates of EGFR and KRAS mutation discordance between primary tumors and BM would be an important contribution to the literature.

Genomic profiling of BM has yielded important information about potentially actionable genomic alterations that may not be detected in the primary tumor, demonstrating that BM can be genetically and phenotypically distinct, in comparison to their primary tumor [15]. Hulsbergen et al., performed a large, multi-institutional study that examined the primary subtype-specific risk of crossover between the primary breast tumor and BM [6]. They found that breast cancer switches subtype in up to 37.5% of BM, with HER2 gain occurs in 14.8% of HER2-negative patients. Similar findings were reported in a recently conducted meta-analysis which showed that breast cancer BM exhibits significant receptor expression discordance in approximately 40% of patients in comparison to primary tumors [7]. Such receptor discordance/subtype switching could have a significant impact on the prognosis and treatment of a patient. These findings could help clinicians decide if acquiring BM tissue might be beneficial in some cases especially when deciding the choice of a targeted treatment.

Given the high intracranial penetration rates of the second and third generation targeted therapies, knowledge of EGFR status is key to biomarker-driven targeted therapy for intracranial disease and awareness of subtype switching is critical for those patients treated with systemic therapy alone for intracranial disease. Wang et al. reported an EGFR T790M mutation in the BM of two patients who had received EGFR TKI treatment, which could be linked to the elevated ploidy levels in these patient BM [16]. EGFR amplification was observed in BM samples but not in lung lesions, suggesting that those patients were resistant to EGFR TKI. Osimertinib a third generation EGFR-TKI has demonstrated excellent CNS response of 91% in patients with EGFR-mutant NSCLC in both first and second-line setting [17]. For example, in the phase III FLAURA trial, the CNS efficacy of osimertinib was demonstrated in the first-line setting with fewer patients in the osimertinib arm developing new brain lesions compared with the control arm (12% versus 30%) [18]. Osimertinib has also shown promising activity in leptomeningeal metastases. The results of this systematic review and meta-analysis show that the EGFR mutation status discordance occurs in about 10% of LCBM, with estimated BM EGFR loss seen in 7% and BM EGFR gain also seen in 7% patients. Knowledge of this could be beneficial especially with regards to patient selection for targeted therapy alone for intracranial disease.

An activating KRAS mutation occurs in approximately 30% of lung adenocarcinomas [19]. It was originally considered an inaccessible target due to the lack of substantial binding pockets for selective small molecule inhibitors. KRAS G12C has emerged as an actionable target for which multiple therapies are under investigation. Recently, sotorasib gained approval for second-line use in patients with metastatic disease harboring the KRAS p.G12C mutation [14]. In our analysis, we found that the KRAS-mutation discordance among LCBM compared to primary tumors was 13%. The estimated KRAS gain in LCBM was 10% and 8% for KRAS loss. Various other agents are under investigation and could pave the way for future therapies to improve outcomes in patients with KRAS mutation. Hence, the knowledge of KRAS receptor discordance between primary and LCBM may help in enhancing outcomes in this subset population in the future.

In this meta-analysis, the mutation assessment techniques varied across the studies, including immunohistochemistry, direct sequencing, high-resolution SNP array, RT-PCR analysis, ARMS method, whole exome sequencing and targeted panel sequencing, and next-generation sequencing (NGS). The differences in each of these assessment techniques potentially will lead to differences in initial detection of these key molecular alterations, however, each study used the same method for the primary tumor and the matched brain metastasis. Therefore, although it is possible that the variation in techniques across studies may lead to some inaccuracy in the assessment of discordance rates, this is mitigated by the use of paired samples. The methods, protocols, the instruments, and the quality of results have evolved considerably during the period of publication of the included studies. Recent guidelines recommend the use of mutant-specific PCR kits, which can usually detect the mutation even if the number of tumor cells in the samples is low [20]. However, some potentially targetable EGFR alterations may still go undetected as none of the currently available PCR kits cover the entire spectrum of EGFR TKI-sensitizing mutations. NGS has the ability to reveal types of EGFR mutations and has a high sensitivity [21]. To yield clearer insights, future studies should perform DNA sequencing studies of the primary tumor and LCBM [22].

Obtaining BM tissue samples for patient management might be challenging, hence non-invasive strategies for analyzing tumor biology and immuno-phenotyping are required [23]. Non-invasive procedures, such as liquid biopsies (circulating tumor cells and cell-free tumor DNA) have recently emerged as a viable detection methods for patients with metastatic lung cancer. Emerging techniques also allow for analysis of cerebrospinal fluid-derived circulating tumor cells (CSF-CTC) and molecular profiling techniques [24]. The best method for detecting the EGFR T790M mutation in the plasma is by using droplet digital PCR [25]. Distinct genomic profiles can be detected by CSF-CTC in leptomeningeal metastases in EGFR mutant NSCLC including increased MET copy number gains and TP53 loss of heterozygosity [26]. EGFR resistant mutations can often be discovered in plasma from NSCLC patients before any clinical symptoms of progression, suggesting that monitoring circulating DNA levels and mutational profiles during the course of the disease could lead to earlier treatment intervention [27, 28]. Advanced imaging and radiomics research could potentially represent a non-invasive approach for predicting tumor immunophenotype, but these approaches are still in an early developmental phase [29].

Till date no model has been developed to predict the LCBM immunophenotype based on patient features and treatment details. However, patient factors such as age, use of systemic therapy, the number and location of sites of metastatic disease are all thought to be associated with receptor expression discordance [7]. We tried to determine the predictors for discordance in EGFR status in patients with LCBM based in individual studies using a meta-regression analysis, but did not find any robust association with any factors. However, some individual studies have shown weak associations between patient factors and change in EGFR status [30–32].

Several evolutionary models, including parallel development and clonal selection have been proposed to explain the discordance rates of EGFR and KRAS between primary tumor and LCBM [33]. The parallel development model describes the discordance seen in synchronous tumors by predicting early generation of disseminated cancer cells to distant organs with highly diverse genetic profiles of the primary and metastasis. The discordance exhibited in metachronous tumors is explained by clonal selection during metastatic spread, with the microenvironment and therapeutic effects potentially having an impact [34]. Concordant events, on the other hand, are likely to be characterized by the same gene model, implying that metastases arise late in the tumor growth process and hence metastatic genetic variation is restricted [35]. During the metastatic process, metastatic relapsing tumors may have acquired new genetic mutations or established resistance (eg. T970M) [36]. However, because the data from individual studies varied, we were unable to show whether the timing of metastases affected the EGFR discordance rates between primary lung and LCBM.

There are several limitations to the present study. First, the results were heterogeneous among the included studies. Second, while there was no evidence of publication bias in this meta-analysis, there is a chance that higher proportions of metastatic-prone immunophenotypes were selected for in each of the individual series in this LCBM-specific meta-analysis. Third, given the retrospective nature of the data collection, technical differences in tumor sample analysis may have contributed to the reporting of pseudo-discordance between the primary tumor and LCBM. Fourth, we did not have the individual patient data of the included studies, preventing assessment of change in receptor status on final treatment outcome. Fifth, mutation assessment technique different and can account for a portion of the variability although this was mostly inter-study variability less than intra-patient. Also, the receptor expression discordance rates could potentially be influenced by tumor heterogeneity and tissue biopsy sample errors.

## Conclusion

In conclusion, the overall discordance rates in EGFR mutation status between primary and LCBM is low. Future researches assessing the impact of EGFR mutation discordance on treatment efficacy and survival are required. Given the high intracranial penetration rates of second and third generation targeted therapies, knowledge of EGFR status is key to biomarker-driven targeted therapy for intracranial disease and awareness of subtype switching is critical for those patients treated with systemic therapy alone for intracranial disease.

## Supplementary Information

Additional file1 (DOCX 376 KB)
