# Supplementary material for: Systematic review and meta-analysis of lung cancer brain metastasis and primary tumor receptor expression discordance
Source: Discov Oncol. 2021 Nov 8;12:48. doi: 10.1007/s12672-021-00445-2 (PMC8777541; doi:10.1007/s12672-021-00445-2)
Supplement: Supplementary file 1 — Additional file1 (DOCX 376 KB) [file 12672_2021_445_MOESM1_ESM.docx]

**Supplemental Figure 1.** PRISMA flow diagram showing the selection of studies for the systematic review of LCBM receptor expression compared to primary tumor immunophenotypes.


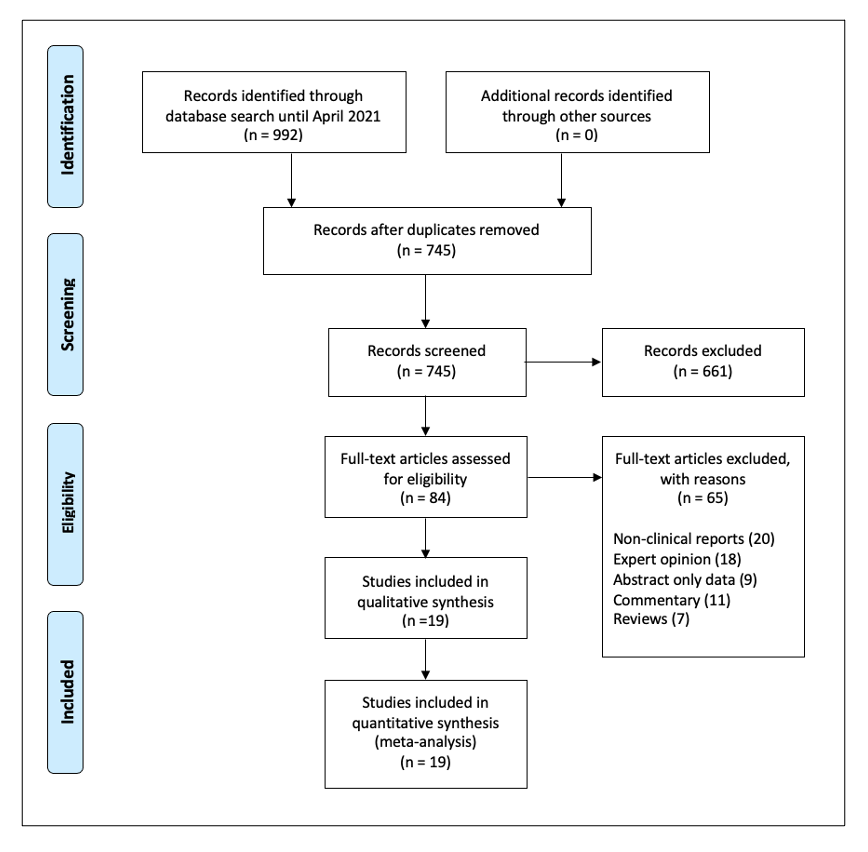


**Supplemental Figure 2**. Funnel plots for (A) for lung/BM EGFR discordance, (B) BM EGFR gain, and (C) BM EGFR loss to assess the potential for publication bias. The measure of effect size and study precision are shown on x- and y-axis.

**
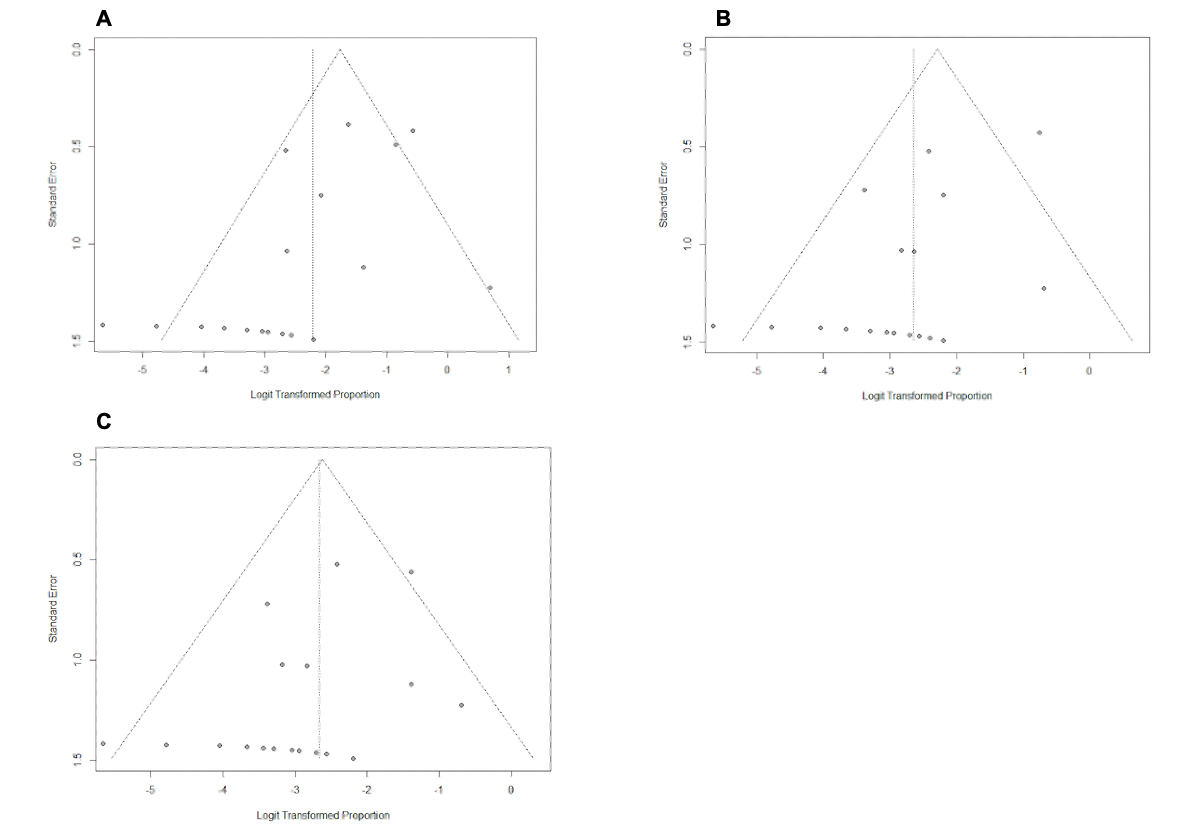
**

**Supplemental Figure 3**. Funnel plots for (A) lung/BM KRAS discordance (B) BM KRAS gain, and (C) BM KRAS loss to assess the potential for publication bias. The measure of effect size and study precision are shown on x- and y-axis.


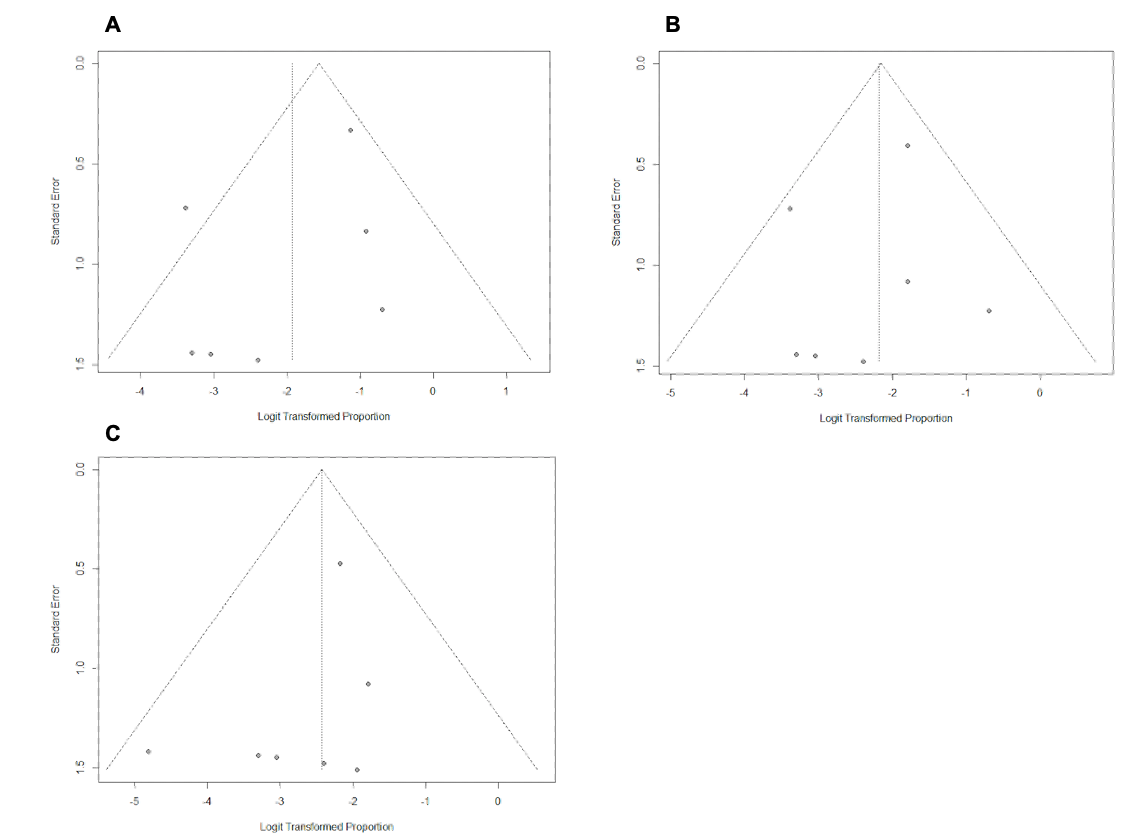


**Supplemental Table 1**: Search strategy for each database

| **Database** | **Search Term** |
| --- | --- |
| **MEDLINE (PubMed)** | (Lung Neoplasms[MeSH] OR lung[tiab] AND (cancer[tiab] OR carcinoma[tiab] OR tumor[tiab]) AND ("Receptor, Epidermal Growth Factor"[MeSH] OR epidermal growth factor receptor[tiab] OR EGFR[tiab]) AND (discordant[tiab] OR discordance[tiab] OR concordance[tiab] OR concordant[tiab] OR conversion[tiab]) |
| **CENTRAL (Cochrane Central Register of Controlled Trials)** | MeSH Term - lung cancer; metastatic; epidermal growth factor receptor; EGFR; discordant; receptor discordance; receptor concordance; receptor concordant; receptor conversion |

**Supplemental Table 2**: PICOS criteria for inclusion and exclusion criteria in meta-analysis

| **Parameter** | **Inclusion Criteria** | **Exclusion Criteria** |
| --- | --- | --- |
| Patient | Patients diagnosed with primary lung tumor and LCBM | Patients with primary lung tumor and extracranial metastases |
| Publication Type | Retrospective or prospective cohort studies >10 patients documenting EGFR and KRAS status in primary lung cancers compared to LCBM, and receptor conversion or discordance published in English. | Reviews, expert opinions, case reports, animal studies, case series <10 patients published in non-English language |
| Intervention | Studies that have tissue samples of primary tumor and LCBM | Studies that have tissue samples of primary tumor and extracranial metastases |
| Outcomes | Studies including the discordance/conversion rates of EGFR and KRAS between primary tumour and LCBM | Studies excluding the discordance/conversion rates of EGFR and KRAS between primary tumour and LCBM |
| Follow-up Time | N/A (pathology comparison study) | N/A (pathology comparison study) |

Abbreviations: LCBM = Lung Cancer Brain Metastases; EGFR = Epidermal growth factor receptor; KRAS = Kirsten rat sarcoma viral oncogene homolog; PICOS = Population, Intervention, Control, Outcomes, Study Design

**Supplemental Table 3 –** GRADE approach to assess quality of the body of evidence

| **Outcomes (number of studies) references** | **Summary of findings** | | **Quality Assessment** | | | |
| --- | --- | --- | --- | --- | --- | --- |
|  | **Number of events** | **Effect size** | **Inconsistency** | **Imprecision** | **Quality** | **Importance** |
| LC / BM EGFR discordance  (19 studies) | 501 | 0.10 (0.05-0.17) | Serious | Serious | Moderate | Important |
| BM EGFR gain  (19 studies) | 501 | 0.07 (0.04-0.12) | Serious | Serious | Moderate | Important |
| BM EGFR loss  (19 studies) | 501 | 0.07 (0.04-0.10) | Serious | Serious | Moderate | Important |
| LC / BM KRAS discordance  (7 studies) | 148 | 0.13 (0.05-0.27) | Serious | Serious | Moderate | Important |
| BM KRAS gain  (7 studies) | 148 | 0.10 (0.06-0.18) | Serious | Serious | Moderate | Important |
| BM KRAS loss  (7 studies) | 148 | 0.08 (0.04-0.15) | Serious | Serious | Moderate | Important |

Abbreviations: LC = Lung Cancer; BM = Brain Metastases; EGFR = Epidermal growth factor receptor; KRAS = Kirsten rat sarcoma viral oncogene homolog
